# Nanotheranostic Application of Fluorescent Protein-Gold Nanocluster Hybrid Materials: A Mini-review

**DOI:** 10.7150/ntno.58060

**Published:** 2021-05-17

**Authors:** Han Ding, Zhijun Chen

**Affiliations:** 1 Wenzhou Institute, University of Chinese Academy of Sciences, Wenzhou, Zhejiang, 325000, China.; 2 State Key Laboratory of Supramolecular Structure and Materials, College of Chemistry and Institute of Theoretical Chemistry, Jilin University, Changchun 130012, China.; 3 Institute for translational medicine, Affiliated Hospital, Medical college of Qingdao University, Dengzhou Road 38, Qingdao 266021, China.

**Keywords:** gold nanoclusters, synthetic method, ion detection, bioimaging, medical applications

## Abstract

The gold nanoclusters (Au NCs) are a special kind of gold nanomaterial containing several gold atoms. Because of their small size and large surface area, Au NCs possess macroscopic quantum tunneling and dielectric domain effects. Furthermore, Au NCs fluorescent materials have longer luminous time and better photobleaching resistance compared with other fluorescent materials. The synthetic process of traditional Au NCs is complicated. Traditional Au NCs are prepared mainly by using polyamide amine type dendrites, and sixteen alkyl trimethylamine bromide or sulfhydryl small molecule as stabilizers. They are consequently synthesized by the reduction of strong reducing agents such as sodium borohydride. Notably, these materials are toxic and environmental-unfriendly. Therefore, there is an urgent need to develop more effective methods for synthesizing Au NCs via a green approach. On the other hand, the self-assembly of protein gold cluster-based materials, and their biomedical applications have become research hotspots in this field. We have been working on the synthesis, assembly and application of protein conjugated gold clusters for a long time. In this review, the synthesis and assembly of protein-gold nanoclusters and their usage in cell imaging and other medical research are discussed.

## Introduction

Gold nanoclusters (Au NCs), a special kind of particles, are usually composed of several to hundreds of gold atoms 1-3, with size approximately several nanometers, which are something between gold atoms and gold nanoparticles. Au NCs show some special properties: such as the surface, quantum size, volume, the red-light quantum tunneling, and the dielectric confinement effects. This phenomenon is mainly attributed to the increase of the local field strength of the Au NC surface and the internal field. The dielectric confinement of Au NCs may have an important effect on optical absorption, optical nonlinearity and photochemistry 4-8.

**Figure 1:**
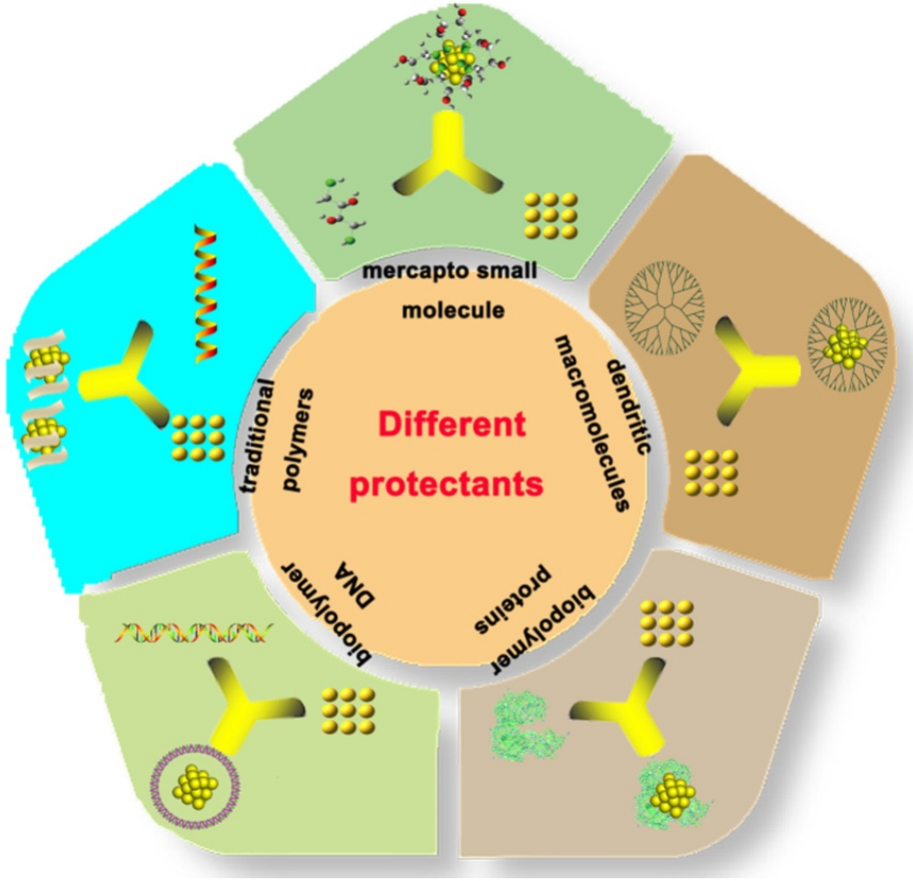
Different strategies for the synthesis of gold nanoclusters.

The Au NCs exhibit unique physical and chemical properties compared with the traditional inorganic light-emitting materials such as quantum dots (QDs) and organic fluorescent molecules. QDs frequently contain heavy metal elements, such as cadmium, lead, and mercury, which are harmful to the human body and the environment. In contrast, organic fluorescent materials have short fluorescence life and display photobleaching property, which hinders their applications in biomedical research. Au NCs contain various advantages such as low toxicity, good biocompatibility, stable luminescence, strong anti-bleaching capability and high stability. Therefore, they display great potential in the field of environmental detection, biomarker development, cell imaging and drug loading 8-24. Different protectants can be used to synthesize Au NCs (Figure 1), such as sulfhydryl small molecule 25, dendritic macromolecules, dksl 26 traditional polymers 27, biopolymer DNA 28, and proteins 29.

Among biomolecules, DNAs and proteins are popular ones used for the synthesis of nanoparticles. DNA has been used to synthesize silver nanoparticles and silver NCs. For instance, Zhou et al. synthesized eight gold Atom-Au NCs by using ultrasonic reactor and DNA etching 28. Liu et al. successfully synthesized Au NCs under acidic conditions by using a single strain DNA as a protectant and two methylamine boranes as a reductant 30. They verified that the luminescence position of the NCs was 725 nm, and the luminescence property was stable.

Many efforts have been conducted on the synthesis of protein capped Au NCs. The sulfhydryl group of cysteine in the protein has a strong affinity with gold, so it serves as a good Au NC stabilizer. Proteins can be cross-linked with the Au NCs. However, Au NCs may locate inside the protein structure, and the protein still preserves its original structure and function 31. It was proposed that under the strong alkaline condition, bovine serum albumin (BSA) could function as the protector and reductant. Xie et al. prepared Au NCs by using BSA 29. They also studied the functional applications of protein-capped gold clusters 32. Besides, many proteins such as lysozyme, pepsin, enoyl-ACP reductase and so on were used to synthesize Au NCs 33-35.

Wei et al. synthesized Au NCs by employing lysozyme as the stabilizer. They managed to diffract the structure of the Lysozyme-Au NCs. Lysozyme-Au NCs were used to study the interaction between protein and gold 36. Liu et al. produced Insulin-Au NCs at low temperature (4 °С) 37. Insulin-Au NCs retains the activity of insulin, which is an example of the original activity containing protein-NCs.

Au NCs synthesized by biomolecules are more biocompatible and harmless than other Au NCs and other materials.

On the basis of the traditional approach of protein-gold cluster synthesis, we used small molecules of mercapto and recombinant protein enoyl-ACP reductase to synthesize gold clusters. By analyzing the differences of gold clusters synthesized using different proteins and comparing their amino acid sequences, we put forward the idea of synthesizing gold clusters based upon protein, amino acid, and chloroauric acid. Therefore, the synthesis of various protein Au NCs hybrid self-assembled materials was tried with BSA and amino acids (e.g., glycine and histidine) in different conditions 38,39. Several gold cluster hybrid materials with different luminescence properties were obtained. The synthesis mechanism of these materials was also elucidated. Based on the results of SDS-PAGE, protein crosslinking occurred in the process of gold cluster synthesis, and free radicals were detected in this process, which may be the cause of crosslinking. We proposed a mechanism of free radicals initiating gold cluster synthesis 39.

## Au NCs synthesized with different proteins

**Figure 2:**
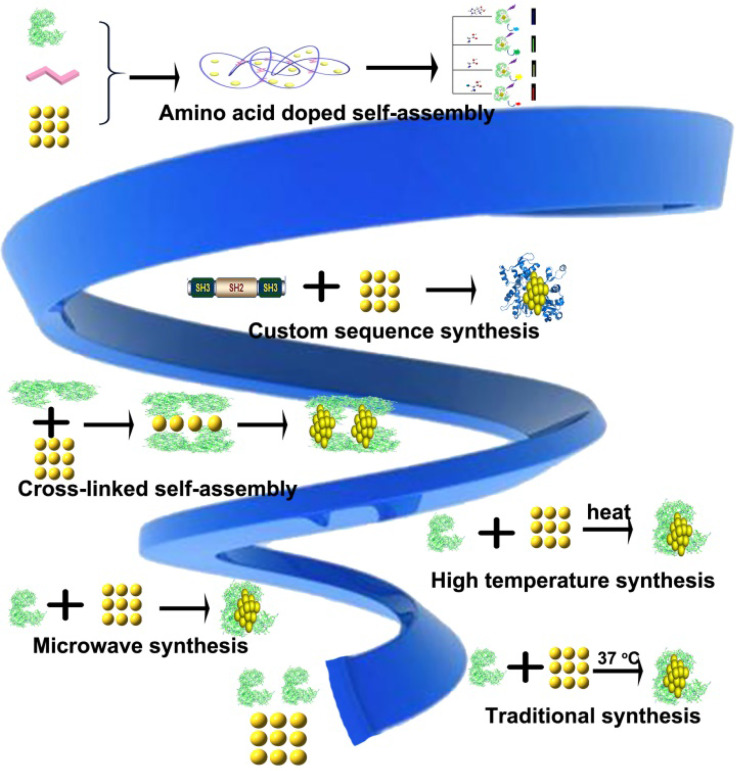
Schematic representation of different approaches for the synthesis of protein-gold nanoclusters.

In 2009, Xie et al. used BSA for the first time to synthesize Protein-Au NCs 29. Thereafter, improved synthetic approaches involving different proteins have been developed to synthesize Protein-Au NCs (Figure 2) 40-51.

### Traditional approach for protein Au NCs synthesis

BSA was frequently used in a traditional method for synthesizing Au NCs. Specifically, BSA and chloric acid were mixed at a certain molar ratio (3:40), and BSA was served as a protector and reducing agent to reduce the trivalent gold to zero value in strong alkaline and 37 °С environment 29. The Au NCs produced by this method usually contains about 25 gold atoms, with size about 1 nm. The prepared Au NCs emitted red fluorescence (640 nm) when excited at 480 nm, the fluorescence quantum yield of which was about 6%.

### Synthesis of Protein-Au NCs by microwave method

Researchers mixed BSA and chloroauric acid at a certain molar ratio (3:40), under strong alkaline followed by microwave treatment 52,53. The Au NCs synthesized by this method contains about 16 gold atoms, the size of which is about 0.8 nm. These AuNCs show red fluorescence (604 nm) with excitation at 365 nm.

### Synthesis of heating protein Au NCs Hydrogel

BSA and chloroauric acid were mixed at a certain molar ratio (25:162) under alkaline conditions. The reaction takes place at 50 °С for 3 hours. The protein was self-assembled to form a gel network structure, which can further reduce the chloroauric acid to form gold nanoclusters. The hydrogel type gold cluster material obtained by this method illuminates at 660 nm under the excitation of 470 nm 54. Scanning electron microscopy (SEM) and transmission electron microscopy (TEM) showed that the whole material exhibited porous fiber structure, with gold embedded in the hydrogel.

### Synthesis of Protein-Au NCs by heating

Researchers further improved the synthetic method. Detailedly, BSA was mixed with chloric acid at a certain molar ratio (3:40), in the strong alkaline and 97 °С. BSA was used as a protective as well as reducing agent to reduce the trivalent gold to zero valent gold; and the gold atoms were coated with BSA to form Au NCs. The size of Au NCs synthesized by this method is about 6.3 nm, with excitation and emission at 375 nm and 625 nm, respectively. Structural characterization showed that several residues of BSA such as tyrosine could be very active in forming radicals under alkali conditions during AuNCs synthesis. These active residues likely triggered radical-mediated cross-linking, which activated the formation of the rod-shaped material 55.

### Other proteins to synthesize protein Au NCs

In addition to using BSA to synthesize protein clusters, researchers also used other proteins to synthesize Protein-Au NCs 55-66.

#### Human serum albumin (HSA)-Au NCs

HSA can be used as a protective agent and reducing agent to synthesize the Protein-Au NCs. HSA was mixed with chloroauric acid at a specific molar ratio (3:40), where HSA served as a protector and reducing agent under strong alkaline and followed by microwave treatment to reduce the valence of the trivalent gold. The synthesized Au NCs gave red fluorescence (655 nm) under the excitation of 368 nm 53.

#### Insulin synthetic protein Au NCs

Insulin and chloroauric acid was dissolved in sodium phosphate, and reacted for 12 h at 4 °С. The Insulin-Au NCs were obtained after centrifuged at 4000 g for 30 minutes. The size of Au NCs synthesized by this method was about 0.92 nm with red fluorescence (670 nm) under 400 nm excitation 37.

#### Lysozyme synthetic protein Au NCs

Lysozyme was also used as a protective agent and reductant to synthesize the protein cluster. Briefly, lysozyme was mixed with chloroauric acid at a certain molar ratio (7:40). Lysozyme served as a protector and reductant to reduce the trivalent gold to zero in a strong alkaline and 37 °С. The size of the resultant Au clusters is about 1 nm, which emitted red fluorescence (657 nm) under 360 nm excitation 33, 36.

#### Lysozyme Type VI synthetic protein Au NCs

The researchers also used lysozyme type VI as the protectant and reductant to synthesize protein-gold clusters. They mixed lysozyme type VI and chloroauric acid according to certain molar ratio (7:50), reacted under strong acid (pH = 3) and 37 °С for 2 days. The Au NCs synthesized by this method emitted 455 nm blue fluorescence under the excitation of 380 nm 67.

If the reaction was performed at 37 °С overnight with pH 12, and a red fluorescent cluster (635 nm) with 380 nm excitation can be obtained 67.

#### Trypsin-Au NCs

Trypsin could also be used as a protective agent and reductant to synthesize the protein Au NCs. Trypsin and chloric acid were mixed in a certain molar ratio (21:125) for 24 h reaction in a strong alkaline and 37 °С. Trypsin was applied as a protector and reductant to reduce the trivalent gold to a zero-valence gold, where and gold atoms were wrapped by trypsin. The size of synthesized Au NCs was about 2 nm, with red fluorescence (640 nm) under 360 nm excitation 68.

#### Pepsin synthetic protein Au NCs

The researchers also used pepsin as a protective agent and reductant to synthesize the protein Au NCs. They mixed pepsin with chloric acid in a certain molar ratio (57:250), after a 12-h reaction in a strong alkaline and 37 °С, using pepsin as a protector and reductant to reduce the trivalent gold to a zero-valence gold. The synthesized Au NCs were wrapped by pepsin. The size of Au NCs synthesized by this method is about 1-2 nm, with red fluorescence (670 nm) under 360 nm excitation 34.

Notably, when pepsin was mixed with chloric acid in a certain molar ratio (57:250), under strong acid (pH=1) and 37 °С for 100-h, the valence of the trivalent gold was reduced to zero-valence. The synthesized Au NCs were wrapped by pepsin, which emitted green fluorescence (510 nm) under excitation (330 nm). Moreover, under alkaline environment (pH=9) and reaction for 24 h, blue fluorescent Au NCs (386 and 456 nm) can be synthesized under the excitation of 330 nm.

Additionally, other research groups have reported the use of other proteins, such as papain, peroxidase and bacterial recombinant protein enoyl-ACP reductase to synthetize Protein-Au NCs 69-75. It is likely that the protein and Au NCs form higher oligomeric complexes through a cross-linking mechanism.

### Using other protein synthetic protein Au NCs and Self-Assembling

#### Self-Assembling Collagen-Gold Hybrid Hydrogel

In acetic acid solution, collagen and chloroauric acid in a certain molar ratio (141:20000) were adequately mixed at room temperature, and the mixture was reassembled overnight to obtain deep red protein gold hydrogels. The size of gold in the hydrogel was between 20 and 70 nm 76. SEM clearly showed that the whole material was a fibrous scaffold structure.

#### Pea protein is loaded (PPI) with gold nanoclusters to self-assemble into composite nanoparticles

First, the PPI was dissolved in a solution containing 6 mol/L quinidine hydrochloride and stirred for 3 hours at room temperature to ensure adequate dissolution. Then, the final concentration of 25 mmol/L dithiothreol was added to the solution. The solution was then dialyzed for two days in a sodium hydroxide solution of pH=10, followed by dialysis in deionized water for one day. The undissolved solids were removed by centrifugation (9000 rpm, 10 min). The pretreated PPI powder was obtained after lyophilization. The pretreated PPI was dissolved in deionized water and mixed with chloroauric acid solution in molar ratio (1:50). Under alkaline condition (pH=13), the gold nanoclusters with a size of about 1 nm were obtained after reaction for 30 minutes at 60 °С. The gold nanoclusters were excited at 635 nm by 480 nm and then the gold clusters were removed from the solution. After full dialysis in ionic water, the pH value gradually returned to neutrality after 3 days of dialysis. In this process, a protein-gold assembly with a size of about 100 nm was formed by self-assembly 77.

### Modular proteins synthetic protein Au NCs

Cortajarena et al. designed consensus tetratricopeptide repeat (CTPR) protein and successfully synthesized stable fluorescent gold nanoclusters using self-designed protein, which laid the foundation for controllable synthesis of different metal nanoclusters 21, 78.

### Protein-gold self-assembled nanomaterials doped with small molecules

**Figure 3:**
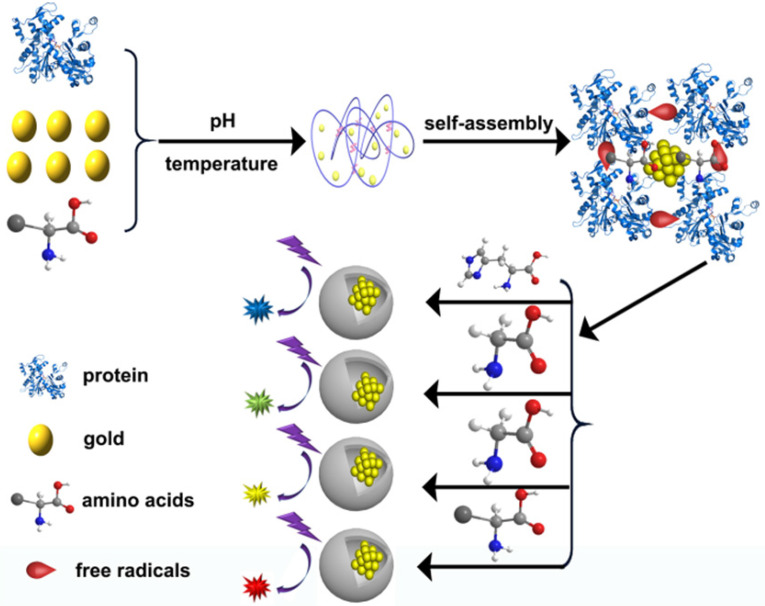
Synthesis of different Protein-Au NCs by amino terminal doping. The reaction process can be considered as self-assembly initiated by free radicals.

Inspired by the above protein synthesis methods, scientists find that the key to protein synthesis is the proportion of amino acids. Therefore, in the process of using BSA synthetic Protein-Au NCs, various kinds of amino acids were doped (Figure 3). Finally, several different kinds of protein doped Au NCs were obtained. Free radicals could be detected from the samples collected during the intermediate process, the reaction mechanism of protein-gold cluster initiated by free radicals was finally determined. Through the results of SDS-PAGE gel, protein crosslinks occurred in the process of gold cluster synthesis, and free radicals were detected in the process of synthesis. We proposed the mechanism of free radicals initiating gold cluster synthesis.

#### Glycine doped synthetic protein-gold self-assembled nanomaterials

In our previous studies, BSA, glycine and chloroauric acid in a certain molar ratio (3:4000:160) were mixed. We adjusted the pH value of the mixed solution. In order to find the most suitable pH conditions for the reaction, we put the reaction under different reaction pH values. We took advantage of pH conditions ranging from 1.5 to 12.5 and thus determined the optimal pH value. Finally, the size of BSA, glycine and chlorofluoric acid was about 1.7 nm. The Au NCs of 515 nm green fluorescence was produced under the excitation of 370 nm. The overall size of the material was about 120 nm. When the BSA and glycine and chlorofluoric acid was synthesized under the condition of pH = 1.5, Au NCs of 560 nm yellow fluorescence were produced with the size of about 1.3 nm, excited by 383 nm. The overall size of the material was about 60 nm. If the reaction was performed with a pH value of 12.5, the size of the material was around 1.8 nm, and the Au NC materials with 607 nm red fluorescence were produced under 372 nm excitation. The overall size of the material was about 100 nm. Besides, referring to the method of synthesizing Au NCs by pepsin, yellow fluorescent materials would be generated when the pH of green fluorescent Au NC material was transferred to 6.8 for 24 h 39.

#### Tryptophan and histidine doped synthetic Protein-gold self-assembled nanomaterials

We mixed the protein BSA, amino acid tryptophan and chlorotic acid in a certain molar ratio (3:4000:160) to adjust the pH value of the mixed solution. We found that the optimal pH value of the reaction was 3. The reaction solution was mixed in a constant temperature at (37 °С) for 12 h. The size of the composite was about 1.8 nm, and the Au NCs of 480 nm blue fluorescence was produced under the excitation of 385 nm. The overall size of the material was about 100 nm 38.

In the same way, we mixed BSA, the group of acid tryptophan and chloroauric acid to a certain molar ratio (3:4000:160) and adjusted the pH value of the mixed solution. The resultant Au NCs (~0.9 nm) of 468 nm blue fluorescence under the excitation of 384 nm were produced under the condition of 5.5. The overall size of the material was about 140 nm 39.

## Study on the application of protein Au NCs

**Figure 4:**
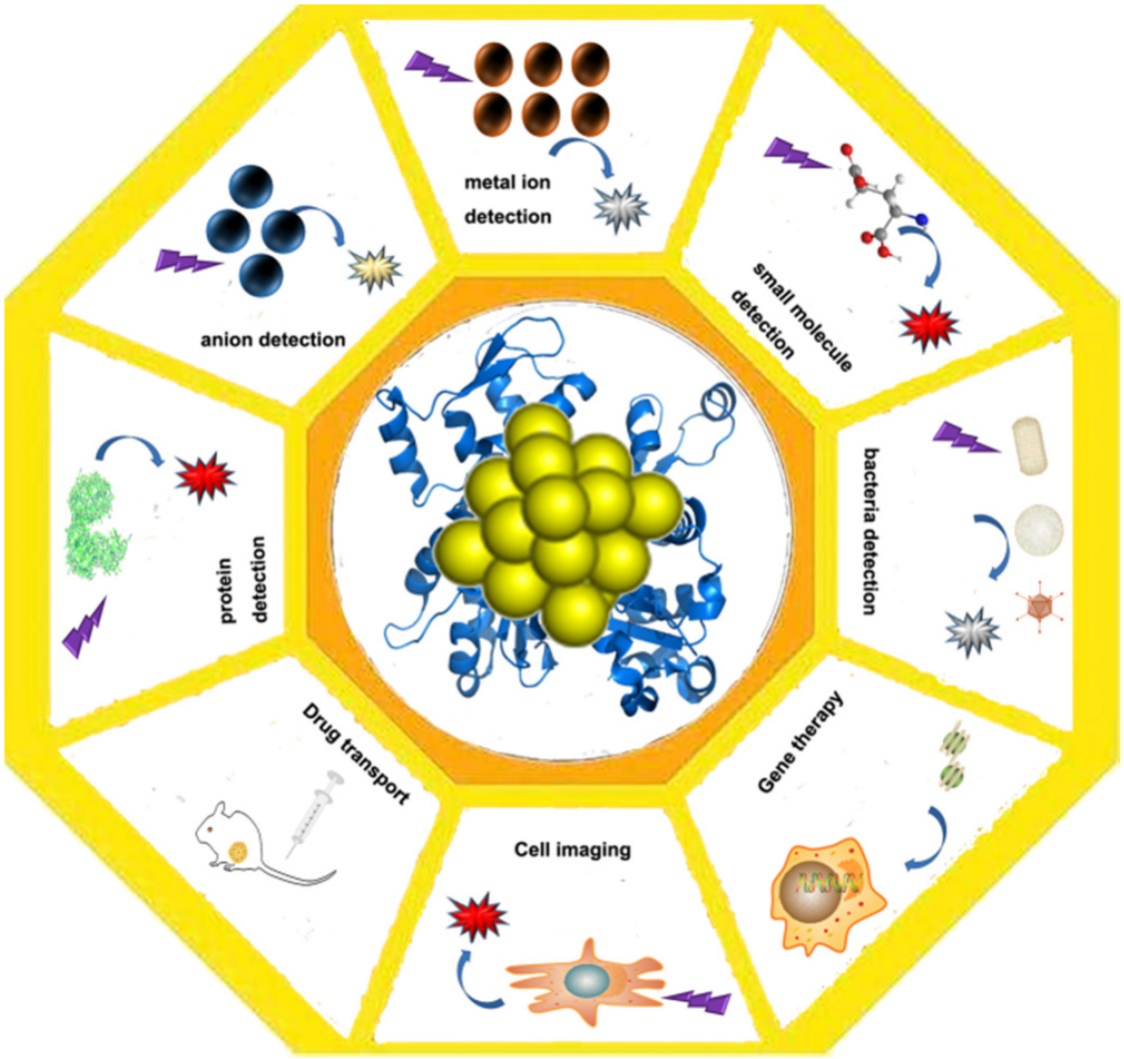
Application of protein-gold nanomaterials. Protein-gold nanomaterials could be used for the detection of ions and small molecules, cell imaging, drug delivery and gene therapy.

As a kind of fluorescent material, Au NCs are mainly used in the detection of some metal ions based on the enhancement and quenching mechanism of their fluorescence. They can be applied for the detection of mercuric ion 79, the detection of the sulfide ions 80, the small organic molecules and the small molecules of the biological molecules, the detection of the biological samples and the trypsin 81, and the application of the fluorescence properties to the effect of the bioimaging 79,82,83. In addition to the use of Au NCs for catalysis, and the application of protein gold derivatives with the development of protein Au NCs hybrid materials (Figure 4), here, we summarize the main applications of protein gold nanoclusters, including the environmental and medical detection of metal ions and other important small molecules and proteins, and t nano diagnosis and treatment including nano imaging, CT imaging and targeted drug delivery.

### Metal ion detection

The detection of heavy metals by Au NCs has also been widely studied and applied. The detection of Hg is the most common, and Hg exists in the environment of soil and water. Hg and its derivatives are mainly accumulated in the nervous system, the digestive system, liver and kidney. Remarkably, Hg can cause damage to respiratory tract mucosa, skin and blood 84. Therefore, many research groups have been dedicated to the detection of Hg ions. Xie et al. used the Protein-Au NCs synthesized by BSA to detect the Hg ion 32. The mechanism involved that the Hg ions specifically caused the fluorescence quenching of the Au NCs. Kawasaki et al. used the Au NCs synthesized by trypsin to detect the Hg ion 68. The Au NCs could detect 20 nM Hg ion. Shang and others used the Au NCs synthesized by DHLA to Hg ion at a very low concentration (0.5 nM) 25. This detection limit was far below the content of Hg ions in drinking water (10 nM). Ding et al. also prepared the Protein-Au NCs for the detection of Hg ion 35. The Protein-Au NCs could be used more conveniently for the detection of Hg ions. The BSA-Au NCs synthesized by Lin et al. could identify the copper ions 85. Copper ions could cause the fluorescence quenching of Au NCs, and they provided the naked eye detection method and the spectrograph detection method. The detection limit reached 10 mM under the naked eye, and the detection limit under the spectrometer could be as low as 0.5 M. In addition, Ding and others used DTT as a protector and reducing agent to quickly synthesize Au NCs, which also identified the copper ions. 86 Copper ions caused the fluorescence quenching of Au NCs, while other metal ions failed to cause the quenching of Au NCs. The detection limit of copper ion was low (80 nM) and the performance was stable. These synthesized Au NCs could be used to effectively detect the copper ions in the serum.

Kawasaki et al. used Au NCs synthesized by pepsin to detect lead ions, and lead ions could enhance the fluorescence of Au NCs 34. Considering that silver ions enhanced the fluorescence of Au NCs, BSA-Au NCs synthesized by microwave method also showed potential in detecting silver ions 52.

### Anion detection

In addition to the detection of metal cations, anions could also be detected by Au NCs. Chen and others used the DNA synthesized to detect sulfur ions, which could be further used for environmental detection 80. The Au NCs may provide protection from sulfide-caused harm to human beings. In addition, Liu and others also tested cyanide by Au NCs, which was a highly toxic pollutant and a great threat to human health 87.

### Small molecule detection

Park et al. used BSA synthetic Au NCs to detect sulfhydryl small molecules glutathione (GSH), cysteine (Cys) and homocysteine (Hcy) 88. The detection mechanism was that the fluorescence quenching of Au NCs could be caused by the aid of Hg ions, and the sulfhydryl group would chelate with the Hg ions to destroy the quenching. The indirect detection of the relationship between three clusters of Au NCs, Hg ions and sulfhydryl molecules were skillfully applied. Dong et al. also used the indirect detection method to detect glucose by using BSA Au NCs 89. The glucose oxidase was used to decompose glucose to produce hydrogen peroxide that quenches the fluorescence of Au NCs. Thus, the effect of glucose was detected. Chen and others used the BSA Au NCs to detect ciprofloxacin 90. They first quenched the Au NCs by copper ions, and then used the carboxyl group on the ciprofloxacin to destroy the Au NCs fluorescence by chelating the copper ions. The BSA Au NCs played a role in the detection of ciprofloxacin 90. Dai et al. also used BSA-Au NCs to detect melamine 91. Melamine was used as an additive in infant milk powder to improve the nitrogen content in milk powder; Dai and others recovered the fluorescence quenching of Au NCs according to the chelating effect of melamine on Hg ions, Au NCs played a role in detection of melamine. Moreover, the detection limits of this method was lower than 0.15 M, far below the national food safety requirement.

### Protein detection

Chen et al. designed Au NCs for protein detection based on the binding principle of glutathione (GSH) and GST in protein purification 92. First, they synthesized the Au NCs protected by GSH, and then used the special interaction between glutathione and GST to detect the GST protein in the solution. Proteins were detected by observing the changes of gold cluster fluorescence with the naked eye or a fluorescence spectrometer.

### Bacteria detection

Chan et al. developed HSA stabilized Au NCs, which was used as a selective luminescence probe for Staphylococcus aureus and methicillin resistant Staphylococcus aureus 93. Also, Ji et al. designed and prepared four kinds of Protein-Au NCs probes for simple, rapid, and accurate identification of bacteria 94.

### Cell imaging application

Wang et al. used BSA-protected Au NCs for identifying specific breast cancer cell SK-BR3, the primary method is to modify Hessaitin (Herceptin) a specific monoclonal antibody against the surface protein of metastatic breast cancer cells on the surface of the BSA Au NCs 95. Shang et al. used the synthetic DHLA-Au NCs for cell imaging 25. The Au NCs were more stable in the cells, be used to test the temperature changes in the cells with Au NCs. Ding et al. also synthesized gold doped cluster materials and used them for cell imaging of fungi and animal cells 38,39, 54,76, 77.

### CT imaging application

Protein-gold nanoclusters not only make use of their luminescent properties for cell imaging, but also have great Stokes potential (Stokes). In recent years, they have also been developed for CT imaging because of its advantages such as displacement and high X-ray absorption efficiency. For instance, Zhang et al synthesized gold nanoclusters using reduced glutathione as template for CT imaging, which has great potential compared with traditional clinical CT imaging 96,97,98.

### Transport of drugs

Hong et al. designed a drug delivery system for gold nanomaterials. When the drug delivery system enters the cell, the drug is released under the action of reduced glutathione in the cell, thus completing the drug delivery 99. Ding et al. used tryptophan doped hybrids to load the drug Adriamycin (Dox) into cells through electrostatic adsorption 38. In the cell, drug release is caused by the weak acidic environment of cancer cells, and drug delivery is completed.

### Gene transport

Li et al. used the hybrids doped by tryptophan to load the plasmids expressing green fluorescent protein into the cells and expressed the green fluorescent protein in the yeast cells 100. Zhang et al. used the DTT protected gold nanoclusters to load the plasmids expressing green fluorescent protein into the cells and expressed the green fluorescent protein in the yeast cells 101. It also opened up a new way for gene therapy by using protein Au NCs instead of viruses.

## Conclusions and outlook

Protein gold nanoclusters are novel nanomaterials with unique physical and chemical properties, which show great potential in many biomedical applications. In the past decade, the research on protein-gold hybrid materials, from the synthetic method to the capping agent selection, has made significant progress. In addition, the study on the characterization of materials has gradually expanded. These researches also focus on the synthesis and in-depth exploration of the synthetic mechanism.

**Figure 5:**
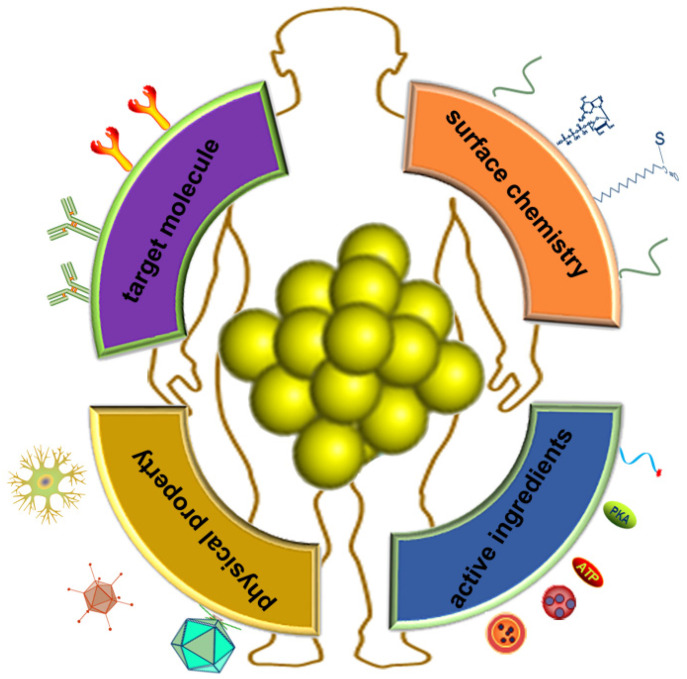
Application prospect of Protein-gold nanomaterials in integration of modification and diagnosis and treatment.

The advantages of fluorescent protein-gold hybrid nanomaterials include simple preparation, environmentally friendly synthesis methods, stable photochemical properties, and low toxicity. In addition, some excellent functions of Au NCs are also elucidated. On the one hand, Au NCs can be used to detect substances from heavy metal ions, small organic molecules and large molecules. The detection method is simple, and the detection performance is stable and the selectivity is high. It is particularly important to develop new probes for detecting global climate change and environmental pollution, especially marine and atmospheric pollution. On the other hand, to cope with the global epidemic such as COVID-19, it's an important direction using protein gold cluster probes for rapid nucleic acid detection, disease diagnosis and reducing diagnostic costs. Besides, the luminescence of protein-nanoclusters is stable, and resistant to the interference such as salt and pH. These Au NCs are also highly biocompatiable and possess decent ant-photo bleaching properties. The complex interactions between nanomaterials and biological systems have attracted great attention of researchers. As a new type of nanomaterials for diagnosis and treatment, protein-gold nanoclusters have shown a lot of potential biomedical applications. Understanding their behavior in the biological environment is an important prerequisite for practical medical applications in the future. Although this field is still in the early stage of research, some researches have clearly shown the significant effects of protein adsorption on the physicochemical properties of gold nanoclusters, and the key roles of protein adsorption, charge interaction and surface ligands on the biological effects of metal nanoclusters 102, 103, 104. Au NCs are important materials for cell imaging in the future. Not only that, Au NCs are also good carriers to carry the necessary ingredients (e.g., small molecule drugs, DNAs and proteins) into target cells (Figure 5). Based on the good drug carrier and modifiable characteristics, further development of protein gold clusters as targeted drugs for tumor and cardiovascular and nucleic acid vaccine has broad application prospects. In order to further understand the behavior of protein capped metal nanoclusters in the biological environment, further research is needed. First, the structure and physicochemical properties of protein-gold nanoclusters are need to be characterized in more detail, so that the effects of specific key parameters can be determined quantitatively. More in-depth and systematic research on protein-gold nanoclusters *in vivo* and their effects on immune system is also the direction of future efforts.
